# Monitoring of polymer type and plastic additives in coating film of beer cans from 16 countries

**DOI:** 10.1038/s41598-021-01723-3

**Published:** 2021-11-11

**Authors:** Nurlatifah, Haruhiko Nakata

**Affiliations:** 1 Graduate School of Science and Technology, Kumamoto University, 2-39-1 Kurokami, Chuo-ku, Kumamoto, 860-8555 Japan; 2 Faculty of Advanced Science and Technology, Kumamoto University, 2-39-1 Kurokami, Chuo-ku, Kumamoto, 860-8555 Japan

**Keywords:** Environmental monitoring, Environmental impact

## Abstract

Plastic debris has gained attention as anthropogenic waste in the environment, but less concerned given to metal waste despite its high abundance in aquatic environment. Metal packaging, such as can, utilizes polymeric coating films as barrier between metals and products which leads to be potential source of microplastic pollution. In this study, 27 beer cans from 16 countries for both body and lid parts as well as inside and outside layers were investigated. Despite the country’s origin, epoxy resin was the major polymeric coating used in all beer cans for lid (inside and outside) and body (inside). Whereas poly(1,2-butanediol isophthalate) was frequently used for outside layer of can body. DEHP and BHT were detected in almost all samples with the highest concentration of 5300 ng/g and 520 ng/g. Despite its lower detection frequency, DOA was detected as high as 9600 ng/g in Belgian beer can. There was no apparent relationship present between the home countries of beer cans and amount of additives used. Despite of being broken down, additives concentration in one environmental sample was found to be one to two orders of magnitude higher compared to the new can. This result proved that adsorption of chemical additives took place in the environment and degraded metal debris may become source of microplastic with higher risk of additives pollution in the environment.

## Introduction

Recently, environmental pollution by marine debris is of great concerns in the world. Previous study summarized the proportion of different types of anthropogenic marine debris on shorelines, and found that plastic was dominant in many sampling sites^1^. Plastic occupied 74, 82, 83, 90, 91 and 84% to total amounts of marine debris in Goto Island, Japan^2^, Wales coast, United Kingdom^3^, Transkei, South Africa^4^, Bootless bay, New Guinea^5^, Midway Atoll, USA^6^, and Chilean coast^7^, respectively. In contrast, metal was also common marine debris in some coastal waters. In contrast, the percentages of total numbers of metal are 40% in N. Devon/Somerset and 37% in Irish Sea in United Kingdom^3^. The metal debris composed 35% and 14% in Fog bay and NE Wales coast in Australia, respectively^8^. The metal cans were the most common drink containers found on these sites.

Similar to coastal environment, the metal debris have been identified in deep-sea. Chiba et.al reported that metal debris was most frequently observed in the North (41%) and South Atlantic (40%) at the depth range of 2300–4935 m^9^. In western North Pacific, more than 500 metal debris were found in the deep sea during 1982–2015. Aluminum beer cans were collected from deep sea of the Ryukyu Trench^10^ and western North Pacific^11^.

Cans have many advantages acting as easy production, storing and transport, and durable^12^. As for consumer, cans are portable, lightweight, temper-resistant, quick-chilling, and stackable^13^. Furthermore, cans, specifically aluminum cans, are highly recyclable material that do not degrade during the recycling process, which makes it the most recycled beverage container in the world. In the United States itself, around 82% of aluminum in used beverage cans enters the recycling system with recycling rate of 50%^14^.

During the manufacturing process, to prevent direct contact between food or beverages and metals that is potentially destructive to each other, polymeric coating films have been widely utilized as they preserve the quality of foodstuff contained within^15^. This polymeric coating films are expected to be biodegradable, having thermal resistance, having high elasticity, having ultimate tensile stress (at least 50%), resistant to abrasion, and having high tightness to water and microorganism with 1 µm to 0.5 mm thickness^12^.

At present, polymer films based on vinyl, acrylic or phenolic-resin are generally used for inner coating^16^. However, epoxy resins based on bisphenol A (BPA) is the most commonly used as coating material^17^. This raised a concern in term of food safety as BPA is a potential endocrine disruptor that imitate the function of estrogen. The average BPA concentration in soft drink products is 0.57 µg/L with daily intake of 0.0034 µg/kg of body weight^18^ which pose less toxic as the Tolerable Daily Intake (TDI) for BPA is 0.01 mg/kg body weight per day^19^.

Polymeric coating film that existed in can packaging may consist of numerous components in the formulation, for instance cross linking agents, catalysts, lubricants, wetting agents, and solvents^17^. In order to increase flexibility of polymer, plasticizers such as dimethyl phthalate (DMP), diallyl phthalate (DAP), dibutyl phthalate (DBP), diisobutyl phthalate (DiBP), benzyl butyl phthalate (BBP), and bis-(2-ethylhexyl) phthalate (DEHP) are generally added^20^. However, recently, restrictions have been brought on the use of phthalate plasticizer especially for DBP, DiBP, BBP, and DEHP^21^ as phthalates additives increase probability of cardiovascular mortality in both adult men and woman^22^. In exchange, other plasticizers, such as dioctyl adipate (DOA), is commonly used as alternative and less toxic plasticizer^23^.

In addition, to stabilize the polymer from degradation due to the presence of UV light or air, antioxidant like butylated hydroxy toluene (BHT) is added in polymer formulation^24^. Due to the weak bonding between plastic additives and polymer^25^ many scientists concern to its leaching ability to food and beverages as well as environment. Phthalates have been reported to be leached to seawater from plastic products from polyethylene bags and polyvinyl chloride cables^26^. However, little information is available on polymer type and plastic additive concentrations in coating film of metal cans produced in the world. Further, polymeric coating film in can debris may be a potential source of microplastic after the deterioration in the environment.

Considering high occurrence of metal debris in marine environment and the low awareness of polymeric coating film in can packaging, this study investigates both polymer type used as lining and the chemical additives contained in plastic film of metal can as further reference for possible threat for marine environment.

## Materials and methods

### Sample collection

In this study, we focused on beer aluminum cans as analytical samples, because they are imported from worldwide and easily purchased in supermarkets and online retailers in Japan. A total number of 27 beer from 16 countries in Asia (China, Indonesia, Japan, Myanmar, Singapore, Thailand, Vietnam), Europe (Belgium, Germany, Spain, Norway, Russia, Sweden, UK), and North America (Mexico and USA) were obtained during September to October 2020 (Table 1). These beers were popular brands as well as domestic craft beers in their home countries, although the bland name could not be mentioned here.

**Table 1:** Origin countries of cans analyzed in this study.

Country	Number of Sample(s)
**Asia (*n *=7)**	(*n *=12)
China	3
Indonesia	1
Japan	3
Myanmar	1
Singapore	1
Thailand	2
Vietnam	1
**Europe (*n *=8)**	(*n *=11)
Belgium	2
Germany	1
Spain	2
Norway	1
Russia	1
Sweden	2
United Kingdom	2
**North America (*n *=2)**	(*n *=4)
Mexico	1
United States of America	3

In addition, one environmental sample of beer can was collected at coastal area of Ariake bay, Kumamoto Prefecture (32^o^53′53.9″ N 130^o^29′14.2″ E) and new can of the same brand were collected for inspection of metal can after weathering condition. The contents of all samples were then emptied and the can packages were washed by water and air dried before analysis.

### Chemicals

In the absence of a clear information about the additives mixture present in can coating. This study focused on plasticizers (including 7 phthalates/PAEs and 1 adipates) and 1 antioxidant that are commonly used in plastic products. These plasticizers are dimethyl phthalate (DMP; CAS# 131-11-3), diethyl phthalate (DEP; CAS# 84-66-2), diallyl phthalate (DAP; CAS# 131-17-9), diisobutyl phthalate (DiBP; CAS# 84-69-5), dibutyl phthalate (DBP; CAS# 84-74-2), benzyl butyl phthalate (BBP; CAS# 85-68-7), di(2-ethylhexyl) phthalate (DEHP; CAS# 117-81-7), dioctyl adipate (DOA; CAS# 123-79-5), and 1 antioxidant (BHT; CAS# 128-37-0). In order to determine the concentrations, six deuterated phthalate standards (*d*_*4*_-DMP, *d*_*4*_-DEP, *d*_*4*_-DiBP, *d*_*4*_-DBP, *d*_*4*_-BBP, *d*_*4*_-DEHP) and deuterated 1-methylnaphthalene were used as surrogate and internal standards, respectively. Detailed information of standard materials is presented on Table S1.

### Analytical procedures

#### Polymer identification

Since the alloy used for body and lid in can packaging are different^14^, this study analyzed both body and lid parts separately to overcome the differences in coating that might be used during the manufacturing process. Body is the part that hold the content within, whereas lid/cover is the part where the can opening is located.

Small portion of each part (approximately 0.5 cm × 0.5 cm) was cut by scissor and polymer type of samples were identified by attenuated total reflectance (ATR) FT-IR (IR Affinity IS, Shimadzu, Japan) for both inside and outside layers. Background spectra was monitored before sample analysis, and isopropyl alcohol (IPA) was used to clean the instrument detector. The FT-IR wavenumber used ranged from 600 to 4000 cm^−1^, and results spectra were then compared with reference library spectra database to determine the polymer type. The quality threshold for polymer identification was a 75% or greater match to the reference library.

#### Chemical additives analysis

Plastic additives were analyzed in both body and lid of each can for all samples. Additionally, one environmental sample and new can of the same brand (body part only) were also analyzed to study the effect of weathering process to chemical additives in can.

Additive analysis was followed by the method reported previously^11^. Fifty to seventy mg of a piece of can was extracted by 1 mL of dichloromethane using ultrasonic instrument for 15 min. Before extraction, 100 ng of deuterated phthalates (*d*_*4*_-DMP, *d*_*4*_-DEP, *d*_*4*_-DiBP, *d*_*4*_-DBP, *d*_*4*_-BBP, *d*_*4*_-DEHP) was spiked into the solution as surrogate. This extraction process was repeated for three times to ensure most of additive chemicals were extracted. After extraction, 200 ng of *d*_*10*_-1-methylnaphthalene was added as internal standard and samples were concentrated until 1 mL under a gentle nitrogen stream. Then 2 µL aliquots were injected into 7890A gas chromatograph coupled to a 5975C mass spectrometer (Agilent Technologies, Santa Clara, CA, USA) with SIM mode. The GC settings and operation condition as well as monitored ions are presented in Supplementary Table S2 and S3. A procedural blank was run in every batch of sample analyses. The recoveries of *d4*-DMP, *d4*-DEP, *d4*-DiBP, *d4*-DBP, *d4*-BBP, *d4*-DEHP were 101 ± 9.0, 103 ± 10, 107 ± 12, 109 ± 13, 125 ± 18, 114 ± 19% respectively. The limits of quantification (LOQ) ranged from 2.1 to 95 ng/g, and the details are shown in Supplementary Table S3.

Statistical analysis was performed by a software of Excel Statistics (Esumi Co. Ltd, Tokyo, Japan). All values used for principal component analysis were standardized by calculating compositions for individual analytes in all can samples.

## Results and discussion

### Polymer types of plastic film in beer cans

**Figure 1:**
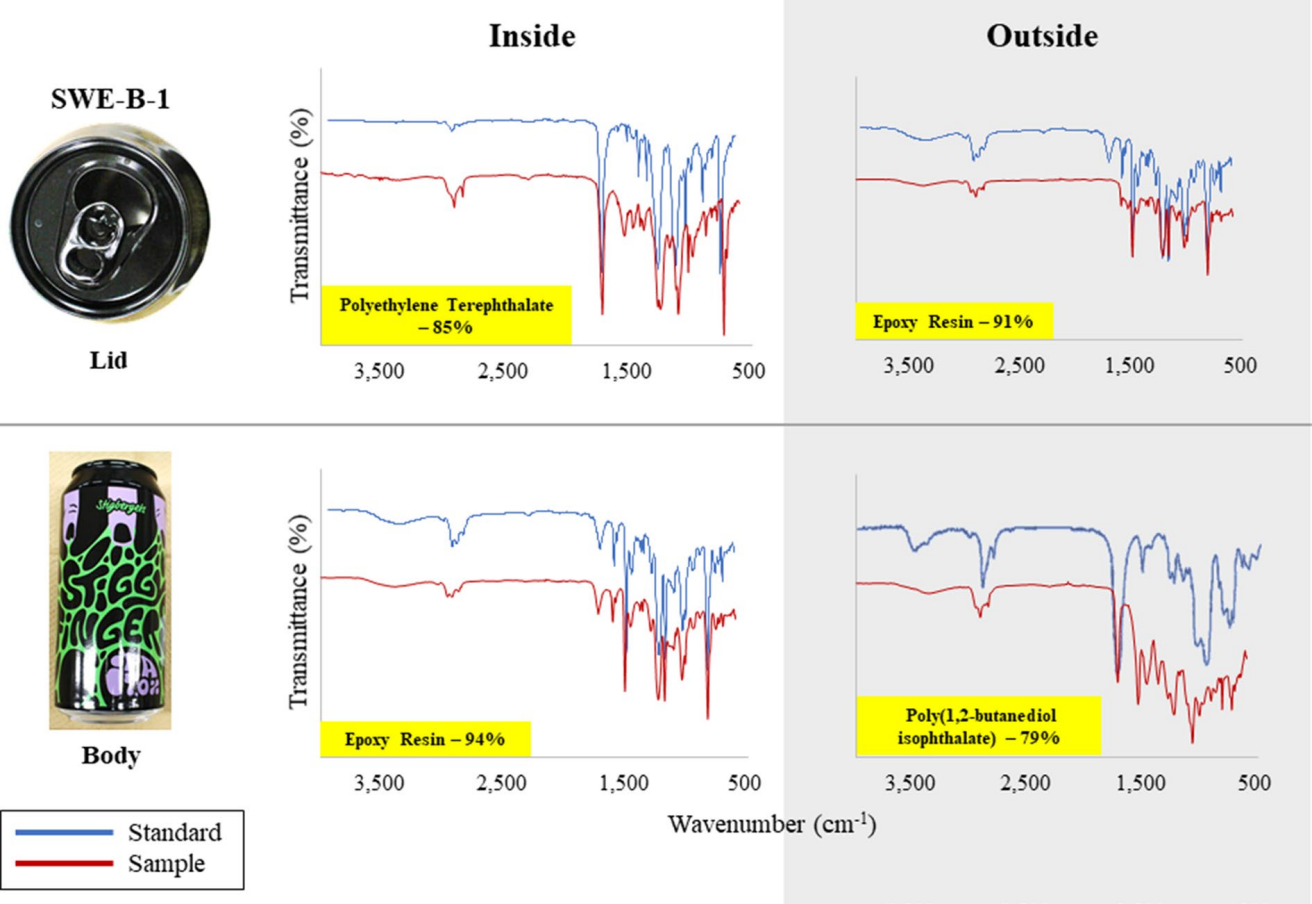
FTIR results for polymer identification in coating film of a beer can.

**Figure 2:**
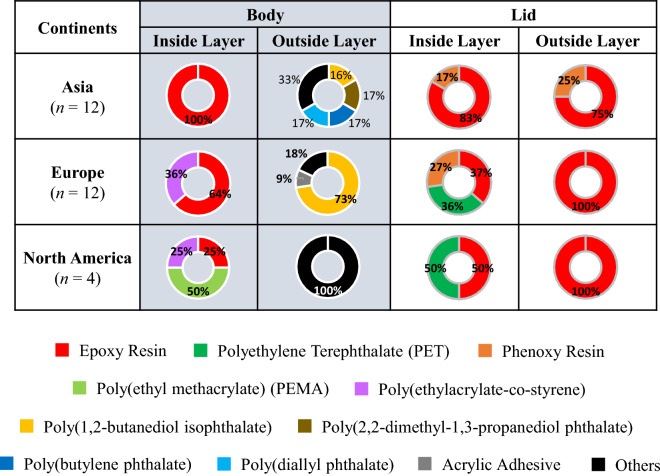
Compositions of polymer type in body and lid of beer cans.

In this study, polymer type of coated part of cans including inside and outside layer for both body and lid were identified. FT-IR results showed that the polymer types used for coating were varied even within the same can. Figure 1 illustrates that in one of samples, SWE-B-1 can, three different polymeric coating were used. Polyethylene terephthalate (PET) and epoxy resin were used in inside and outside coating of lid, whereas for inside and outside coating of body, epoxy resin and poly(1,2-butanediol isophthalate) were used respectively. Apart from the country origin of cans, epoxy resin was the prevailing polymeric coating used in both body and lid in all cans analyzed (Fig. 2, Table S4). Especially, inside layer of body was coated by epoxy resin in all Asian beer cans (*n* = 12). Epoxy resin is commonly used in can coating as it features firmness to heat condition, adhesion, formability, chemical resistance under many conditions^17^. Not to mention it also flexible and adhere well to different metal surfaces^27^. Epoxy resin was also frequently used in inside layer of North American and European beers bodies, followed by poly(ethyl methacrylate) and poly(ethylacrylate-co-styrene) (Fig. 1, Table S4). As some printing exhibited in almost all surface of the outside coatings of can body, the polymeric coating used were more varied with poly(1,2-butanediol isophthalate) being the most frequently employed in European beer cans (Fig. 1).

The lid part used different type of coating materials with epoxy resin was the commonly used especially for outside layer (Fig. 1). While the inside layer employed not only epoxy resin but also PET in European and North American cans and phenoxy resin in Asia and European cans (Fig. 1, Table S4). Due to various concern to BPA, other polymeric coating materials have been introduced to the market, such as polyester and acrylic-phenolic materials^28^. Phenolic resins made from phenols and aldehydes are highly corrosion resistant and have less flexibility properties^27^.

### Chemical additives contained in beer cans

**Figure 3:**
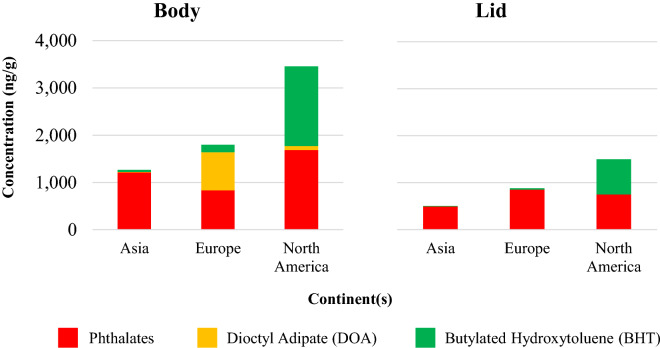
Additive concentrations in coating film of body (left) and lid (right) of beer cans.

**Figure 4:**
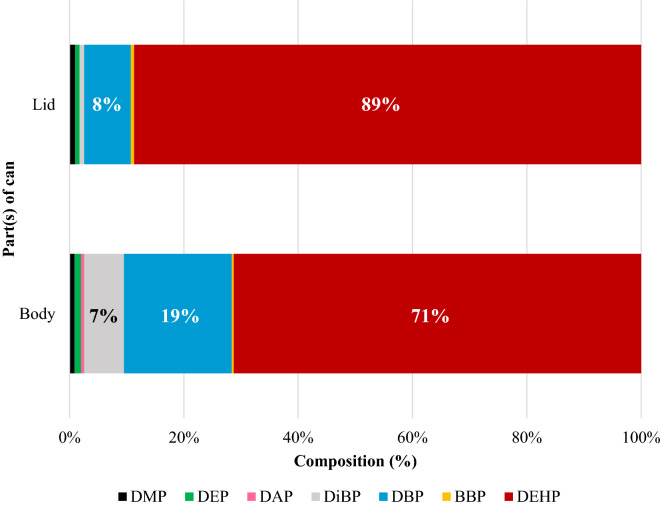
Composition of phthalate additives concentrations in body and lid of beer cans.

Both body and lid part of all cans were subjected to analyzed. In general, the concentrations of additives in body part were higher than those in lid part (Fig. 3). Additive’s composition in both body and lid were dominated by phthalate additives (PAEs) followed by BHT. DEHP was used as the major PAE additive used in can coatings (Fig. 4), occupying for 71% and 89% both body and lid respectively and followed by DBP as the second frequently used PAE additive. At least an average of 450 ng/g of DEHP (Table 2) was detected beer can with the highest concentration of DEHP was detected in lid part of Norwegian beer for 5300 ng/g (Table S5). Both body and lid parts utilized the same average concentration of DMP and DEP. Whereas for DiBP, DBP, and BHT, body parts utilized one order magnitude higher in concentration than lid part. DEHP, DBP, DiBP, and BBP are actually the four phthalates candidate for substance of very high concern (SVHC) that required authorization prior using^21^. It was interesting to know that all analytes except for DEHP and BHT were not detected in UK-B-2 can, although this can utilized the same polymer coating with UK-B-1.

**Table 2:** Plastic additives concentration (ng/g) by continents in beer cans.

Continents	Criteria	DMP	DEP	DAP	DiBP	DBP	BBP	DEHP	DOA	BHT
**Body**										
Asia (*n *=12)	Mean	12	17	0	77	324	44	788	15	45
	Median	7.5	8.0	0	52	210	0	575	0	22
	Min–max	5.0–23	0–60	0	0–310	0–1500	0–110	0–1900	0 -48	6.0–190
	Detection frequency (%)	100	50	0	92	75	8.3	83	33	100
Europe (*n *=11)	Mean	8.1	8.0	36	85	145	0	506	880	165
	Median	9.0	0	0	22	120	0	510	0	150
	Min–max	0–12	0–33	0–120	0–290	0–320	0	0–1000	0–9600	18–410
	Detection frequency (%)	91	27	9.1	73	64	0	82	18	100
North America (*n *=4)	Mean	6.8	6.8	0	33	101	0	1,565	85	298
	Median	6.5	0	0	35	60	0	1,550	14	275
	Min–max	5.0–9.0	0–22	0	0–62	0–190	0	860–2300	0–300	120–520
	Detection frequency (%)	100	25	0	75	50	0	100	50	100
**Lid**										
Asia (*n *=12)	Mean	6.8	6.5	0	6.0	66	0	450	5.7	9.1
	Median	6.5	0	0	0	0	0	325	0	7.5
	Min–max	0–13	0–20	0	0–17	0–160	0	0–1700	0–12	0–19
	Detection frequency (%)	92	33	0	42	25	0	92	8.3	92
	Mean	6.2	6.0	0	6.4	52	45	722	0	28
Europe (*n *=11)	Median	5.0	0	0	0	0	0	270	0	11
	Min–max	0–10	0–23	0	0–20	0–99	0–110	0–5300	0	0–110
	Detection frequency (%)	91	27	0	36	9.1	9.1	73	0	91
North America (*n *=4)	Mean	7.8	7.3	0	0	0	0	735	0	91
	Median	7.5	0	0	0	0	0	590	0	65
	Min–max	6.0–10	0–24	0	0	0	0	360–1400	0	15–220
	Detection frequency (%)	100	25	0	0	0	0	100	0	100

*DMP* dimethyl phthalate, *DEP* diethyl phthalate, *DAP* diallyl phthalate, *DiBP* diisobutyl phthalate, *DBP* dibutyl phthalate, *BBP* benzyl butyl phthalate, *DEHP* bis(2-ethylhexyl) phthalate, *DOA* dioctyl adipate, *BHT* butylated hydroxytoluene.

An antioxidant of BHT was also detected in almost all samples (ranged from 6.0 to 520 ng/g) with highest average concentration was detected in North American beer for 298 ng/g (Table 2). During manufacturing process, antioxidant is commonly added to protect polymer from undergo oxidation mechanism^23^ due to illumination and mechanical stress^29^. On the other hand, DOA was only frequently detected in the body part (Table 2) and in lid part of THA-B-2 samples (Table S5). Moreover, Body part of BEL-B-2 sample was detected to contain the highest concentration of DOA for 9600 ng/g (Table S5). Adipates have actually been demonstrated to have greater solubility in polar solvents such as 3% acetic acid and 10–35% ethanol^30^. DAP and BBP were only detected in few samples (Table 2).

**Figure 5:**
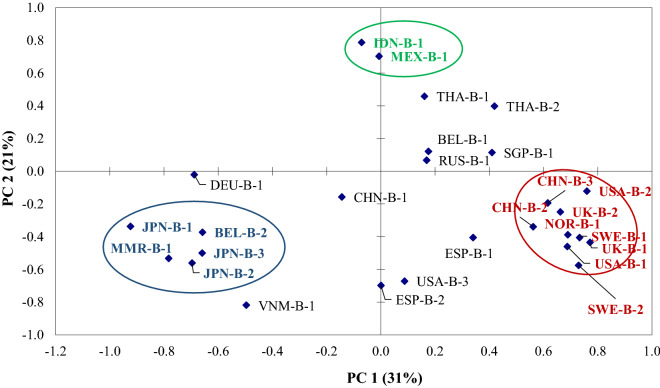
Principal component analysis (PCA) to the plastic additive compositions in beer cans (DMP, DEP, DiBP, DBP, DEHP, DOA, and BHT). The eigenvalues of PC1 and PC2 are described in Table S7.

A principal component analysis of 5 PAEs, DOA and BHT was performed for exploring the similarities or differences between samples. There were two principal components extracted which explaining 31% and 21% of the total variance for PC 1 and PC 2 respectively (Fig. 5). The dominant eigenvalues were DEHP, BHT, and DEP for PC1 and DOA and DEP for PC2 (Fig. 5, Table S6). The graphic distribution showed that there were at least three groups of beer can having the same characteristic in additives concentration. Some European, North American and two Chinese beer cans (marked in red color) have same characteristic with high concentration of both DEHP and BHT. Whereas Indonesian and Mexican beer cans (marked in green color) shared the same characteristic having high concentration of DOA. One intriguing result was that all Japanese beer analyzed in this study, along with Myanmar beer can and one Belgian beer can (marked in blue color) shared similarities in containing high concentration of DEP (Fig. 5). However, there were no particular pattern and correlation between additives concentration and beer can origin observed in this study. This result suggests that the manufacturing country of beer cans might be different from the country where these products were being marketed.

### Investigation on deteriorated sample

This study suggested that wide variety of plastic polymers are used in films of inside/outside and body/lid of beer cans. As described earlier, metal debris including cans have been found in marine debris in both coastal and deep-sea environment, but previous studies categorized can as ‘metal’, not plastic. Recently, Nurlatifah et al.^11^ analyzed plastic film of a Chinese beer can from the western North Pacific (depth: 5813 m) which were found remained intact. The types of polymer are epoxy resin in inside and poly(triethyleneglycol isophthalate) in outside of the can body.

**Figure 6:**
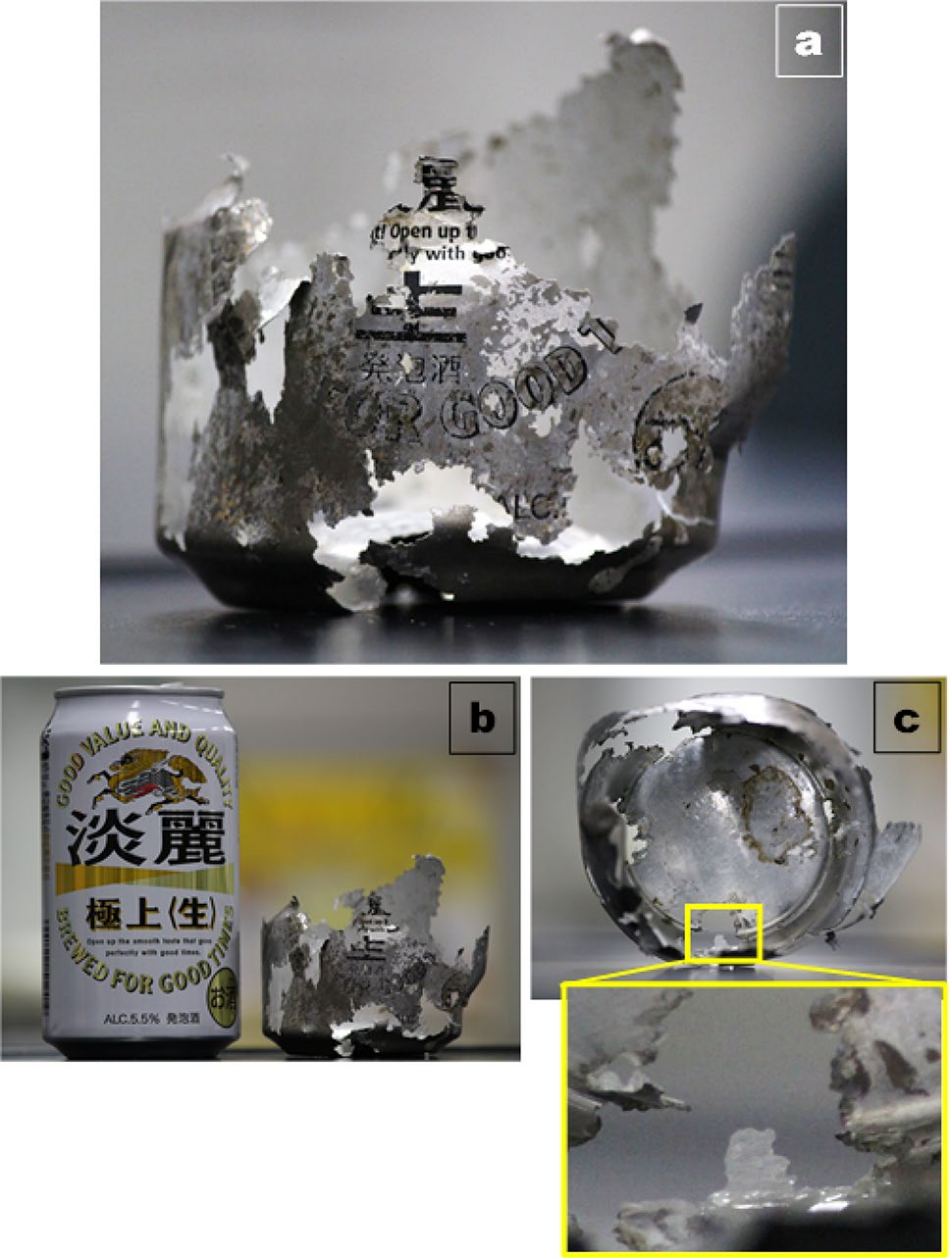
**(a)** Pictures showing a deteriorated can collected from Ariake bay in Japan, **(b)** comparison of external conditions between deteriorated and new can, **(c)** plastic coated film being visible due to deterioration process.

On the other hand, deterioration process was observed in one field sample found in the beach in Ariake bay (Fig. 6a). This field sample was found with only half of can body remained (Fig. 6b). Although the outside coating film was hardly remained, the inner coating was still well preserved and being peeled out (Fig. 6c). This suggest that the outside coating is more prone to breakdown and release microplastic to environment. FT-IR results proved that degradation was hardly taken place for inner coating of field sample as there were no changes in FT-IR spectra compared to the new one (Fig. S1). In contrast, the outside coating seemed to undergo degradation process, proven by changes in FT-IR spectra of field sample which was far different from the new one.

In pursuance of understanding its toxicological risk, additives analysis was performed for both field and new can samples. An intriguing result showed that aging can contained a higher concentration of additives for more than one order of magnitude (Table 3). The new can only contained DEHP and DOA as plasticizer and BHT as its antioxidant at the levels of 1600 ng/g and 61 ng/g, respectively. Whereas the can retrieved from the environment contained other PAEs additives such as DMP, DEP, DiBP, and DBP. Liu et al.^31^ also reported that ionic strength such as NaCl and CaCl_2_ can promote the sorption of DBP and DEP in microplastics of PS, PE, and PVC due to the salting out effect. This result shows that during the weathering process, additives may not only leach to the environment, but also adsorb to the surface of the polymer.

**Table 3:** Comparison of additives concentration in can body of field sample to new can.

Target analyte(s)	Additives concentration (ng/g)	
	Field sample	New can
DMP	3.7	<2.9
DEP	45	<3.6
DAP	<55	<55
DiBP	120	<2.1
DBP	450	<95
BBP	<76	<76
DEHP	1600	1100
DOA	61	20
BHT	14	6.2

In contrast, a metal can, having the same brand with this study but retrieved from the sea floor of the West Pacific, only contained DMP and BHT with lower concentration of 9.0 ng/g and 18 ng/g respectively^11^. The migration rate or leaching ability of polymeric coatings may vary according to polymer thickness^23^, but the possibility of chemical additives to leach to surrounding environment has gained attention due to its weak bond to the polymer^25^. Paluselli et al.^26^ reported the ability of phthalates to leach to seawater from plastic products as much as 120 ng/g, 83 ng/g, 69 ng/g, and 9.5 ng/g for DBP, DiBP, DEP, and DMP respectively from polyethylene bags and polyvinyl chloride cables. Other study reports how the leachate from plastics may inhibit marine microbes^32^.

## Conclusions

In this study, 27 beers cans from 16 countries were analyzed to understand polymer type of their coating materials. Epoxy resin was the major polymeric coating used in all beer cans for both inside and outside layers of the lids and inside layer of the bodies. As for the outside layer of can body, poly(1,2-butanediol isophthalate) was frequently used. Additive chemicals contained in both body and lid part of beer cans were also analyzed. DEHP was detected in almost all samples with the highest concentration of 5300 ng/g. Whereas BHT was detected in body parts of all cans and almost in all lid parts of cans with maximum concentration of 520 ng/g. Despite its lower detection frequency, DOA was detected as high as 9600 ng/g in Belgian beer can. Overall, there were no particular pattern of additives concentration and manufacturing countries of beer can itself. One deteriorated environmental sample was found to contain one to two orders of magnitude higher concentration of additives compared to the new can. Considering the high occurrence of metal debris in marine environment after plastic debris, it is important to understand that materials of can are both metal and plastic, and it becomes a potential source of microplastic in the marine environment after breaking down. Moreover, the microplastic and additives originated from metal cans need to be monitored their potential adverse effects to aquatic ecosystem.

## Supplementary Information

Supplementary Information.

## Abbreviations

BPA: Bisphenol A
DMP: Dimethyl phthalate
DEP: Diethyl phthalate
DAP: Diallyl phthalate
DBP: Dibutyl phthalate
DiBP: Diisobutyl phthalate
BBP: Benzyl butyl phthalate
DEHP: Bis-(2-ethylhexyl) phthalate
DOA: Dioctyl adipate
BHT: Butylated hydroxy toluene
PAEs: Phthalates
ATR FT-IR: Attenuated total reflectance Fourier-transform infrared
IPA: Isopropyl alcohol
SIM: Selected ion monitoring
GC: Gas chromatography
LOQ: Limit of quantification
PET: Polyethylene terephthalate
SVHC: Substance of very high concern
