# Supplementary material for: Monitoring of polymer type and plastic additives in coating film of beer cans from 16 countries
Source: Sci Rep. 2021 Nov 11;11:22115. doi: 10.1038/s41598-021-01723-3 (PMC8586161; doi:10.1038/s41598-021-01723-3)
Supplement: Supplementary file 1 — Supplementary Information. [file 41598_2021_1723_MOESM1_ESM.pdf]

Supplemental files

Monitoring of polymer type and plastic additives in coating film of beer cans from 16 countries

Nurlatifah[1)] and Haruhiko NAKATA[2)]*

1) Graduate School of Science and Technology, Kumamoto University, 2-39-1 Kurokami, Chuo-ku, Kumamoto 860-8555, Japan.
2) Faculty of Advanced Science and Technology, Kumamoto University, 2-39-1 Kurokami, Chuo-ku, Kumamoto 860-8555 Japan.

*: Corresponding authors
Email address: nakatah@kumamoto-u.ac.jp (Haruhiko Nakata)

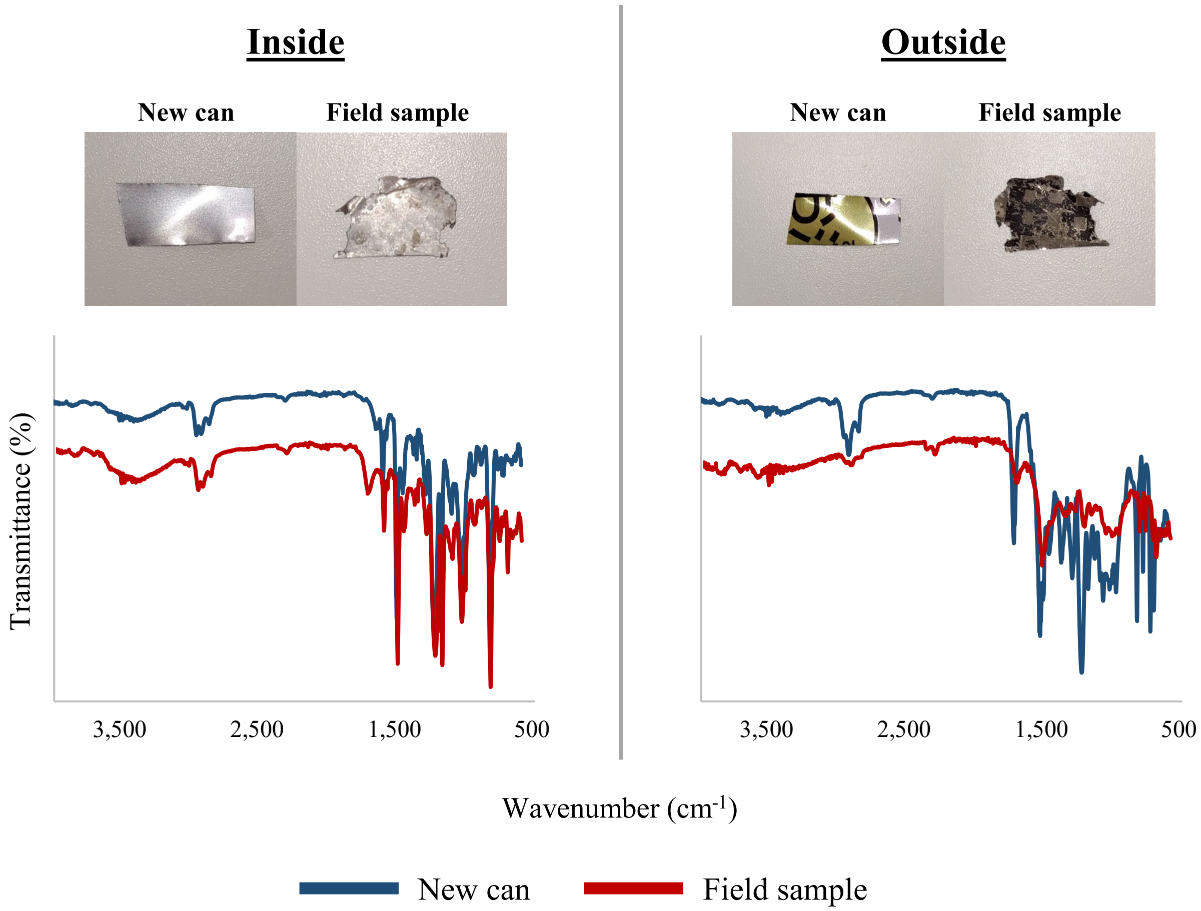

Fig S1. Comparison for FTIR result of field sample and new can of the same brand for inside (left) and outside (right) coating film

Table S1. Information on target compounds and standards

Synonyms	Chemical Name	CAS No.	Supplier	Purity (%)	Log Kow
**Plasticizer(s)**					
DMP	Dimethyl phthalate	131-11-3	Toronto Research Chemicals	98	1.66
DEP	Diethyl phthalate	84-66-2	Toronto Research Chemicals	97	2.65
DAP	Diallyl phthalate	131-17-9	Tokyo Chemical Industry Co. LTD	98	3.36
DiBP	Diisobutyl phthalate	84-69-5	Toronto Research Chemicals	98	4.46
DBP	Dibutyl phthalate	84-74-2	Toronto Research Chemicals	98	4.61
BBP	Benzyl butyl phthalate	85-68-7	Toronto Research Chemicals	98	4.84
DEHP	Bis(2-ethylhexyl) phthalate	117-81-7	Toronto Research Chemicals	98	8.39
DOA	Dioctyl adipate	123-79-5	Fujifilm-Wako Pure Chemical Corporations	99	8.26
**Antioxidant**					
BHT	Butylated hydroxytoluene	128-37-0	Cayman Chemical Company	98	5.03

Table S2. Instrumental condition of GC-MS

GC	Agilent Technologies 7890A
Detector	Agilent Technologies 5975C VL MSD
Carrier gas	Helium (99.999%)
Inlet settings:	
Inlet temp.	270°C
Pressure	19.274 psi
Septum purge flow mode	Standard
Septum purge flow	3 mL/min.
Injection mode	Splitless
Purge flow to split vent	50 mL/min. at 1 min.
Gas saver	20 mL/min. after 2 min
Column	Agilent HP-5MS: 325°C: 30m x 250μm (id) x 0.25μm (ft)
Flow	1 mL/min
Pressure	9.3825 psi
Oven settings:	
Oven temp. program	80°C (1 min.), 20°C/min., 160°C (0 min.), 3°C/min., 300°C (15 min.)
Run time	66.67 min.
Equilibration time	0.5 min.
Post run	50°C, 0 min.
MS transfer line	280°C
Detector settings:	
Ion source temp.	230°C
Scan mode	SIM
Start time	2 min.

Table S3. Information on retention times, fragment ions (for quantification), and LOQ values of target compou

Synonyms	Chemical Name	Retention Time (min.)	Monitored Ions (m/z)	LOQ Concentration in Plastic (ng/g)
**Plasticizer(s)**				
DMP	Dimethyl phthalate	7.20	163, 194	2.9
DEP	Diethyl phthalate	9.01	149, 177	3.6
DAP	Diallyl phthalate	11.71	149, 189	55
DiBP	Diisobutyl phthalate	14.25	149, 223	2.1
DBP	Dibutyl phthalate	16.47	149, 223	95
BBP	Benzyl butyl phthalate	26.33	149, 206	76
DEHP	Bis(2-ethylhexyl) phthalate	31.37	149, 167	58
DOA	Dioctyl adipate	27.68	129, 112	10
**Antioxidant(s)**				
BHT	Butylated hydroxytoluene	7.91	205, 220	4.5

Table S4. Detailed information and polymer type of coating on aluminium cans analysed in this study

Sample Code	Country	Polymer Type			
		Body		Lid	
		Inside Layer	Outside Layer	Inside Layer	Outside Layer
**Asia (*n* = 12)**					
CHN-B-1	China	Epoxy Resin	Poly(diallyl phthalate)	Epoxy Resin	Epoxy Resin
CHN-B-2	China	Epoxy Resin	Poly(2,2-dimethyl-1,3-propanediol isophthalate)	Epoxy Resin	Epoxy Resin
CHN-B-3	China	Epoxy Resin	Poly(butylene phthalate)	Epoxy Resin	Epoxy Resin
IDN-B-1	Indonesia	Epoxy Resin	Poly(butylene phthalate)	Epoxy Resin	Epoxy Resin
JPN-B-1	Japan	Epoxy Resin	Poly(esterurethane)	Epoxy Resin	Epoxy Resin
JPN-B-2	Japan	Epoxy Resin	Poly(hexamethylenemaleate-co-styrene)	Epoxy Resin	Epoxy Resin
JPN-B-3	Japan	Epoxy Resin	Poly(1,3-propanediol isophthalate)	Epoxy Resin	Epoxy Resin
MMR-B-1	Myanmar	Epoxy Resin	Poly(2,2-dimethyl-1,3-propanediol phthalate)	Epoxy Resin	Phenoxy Resin
SGP-B-1	Singapore	Epoxy Resin	Poly(2,2-dimethyl-1,3-propanediol phthalate)	Epoxy Resin	Phenoxy Resin
THA-B-1	Thailand	Epoxy Resin	Poly(1,2-butanediol isophthalate)	Phenoxy Resin	Epoxy Resin
THA-B-2	Thailand	Epoxy Resin	Poly(1,2-butanediol isophthalate)	Epoxy Resin	Phenoxy Resin
VNM-B-1	Vietnam	Epoxy Resin	Poly(diallyl phthalate)	Epoxy Resin	Epoxy Resin
**Europe (*n* = 11)**					
BEL-B-1	Belgium	Epoxy Resin	Poly(1,2-butanediol isophthalate)	Epoxy Resin	Epoxy Resin
BEL-B-2	Belgium	Epoxy Resin	Poly(1,2-butanediol isophthalate)	Phenoxy Resin	Epoxy Resin
DEU-B-1	Germany	Poly(ethylacrylate-co-styrene)	Poly(propyleneglycol isophthalate)	Polyethylene Terephthalate	Epoxy Resin
ESP-B-1	Spain	Poly(ethylacrylate-co-styrene)	Acrylic Adhesive	Phenoxy Resin	Epoxy Resin
ESP-B-2	Spain	Epoxy Resin	Poly(2,3-diethyl-1,3-propanediol isophthalate)	Epoxy Resin	Epoxy Resin
NOR-B-1	Norway	Epoxy Resin	Poly(1,2-butanediol isophthalate)	Epoxy Resin	Epoxy Resin
RUS-B-1	Russia	Epoxy Resin	Poly(1,2-butanediol isophthalate)	Epoxy Resin	Epoxy Resin
SWE-B-1	Sweden	Epoxy Resin	Poly(1,2-butanediol isophthalate)	Polyethylene Terephthalate	Epoxy Resin
SWE-B-2	Sweden	Epoxy Resin	Poly(1,2-butanediol isophthalate)	Phenoxy Resin	Epoxy Resin
UK-B-1	United Kingdom	Poly(ethylacrylate-co-styrene)	Poly(1,2-butanediol isophthalate)	Polyethylene Terephthalate	Epoxy Resin
UK-B-2	United Kingdom	Poly(ethylacrylate-co-styrene)	Poly(1,2-butanediol isophthalate)	Polyethylene Terephthalate	Epoxy Resin
**North America (*n* = 4)**					
MEX-B-1	Mexico	Epoxy Resin	Poly(methacrylic acid ester)	Epoxy Resin	Epoxy Resin
USA-B-1	United States of America	Poly(ethyl methacrylate)	Poly(1,5-pentanediol adipate-co-isophthalate)	Epoxy Resin	Epoxy Resin
USA-B-2	United States of America	Poly(ethyl methacrylate)	Poly(propyleneglycol isophthalate)	Polyethylene Terephthalate	Epoxy Resin
USA-B-3	United States of America	Poly(ethylacrylate-co-styrene)	Poly(methylmethacrylate-co-vinylidenechloride)	Polyethylene Terephthalate	Epoxy Resin

Table S5. Concentrations of plastic additives (ng/g) in body and lid of beer cans analyzed in this study

Sample Code	Plasticizer(s)								Antioxidant
	DMP	DEP	DAP	DiBP	DBP	BBP	DEHP	DOA	BHT
**Body**									
**Asia (*n* =12)**									
CHN-B-1	7.0	16	<55	81	380	<76	1500	<10	22
CHN-B-2	6.0	<3.6	<55	11	<95	<76	330	<10	11
CHN-B-3	17	<3.6	<55	19	190	<76	1900	<10	16
IDN-B-1	6.0	<3.6	<55	310	530	110	1100	45	39
JPN-B-1	21	60	<55	190	330	<76	<58	24	74
JPN-B-2	18	34	<55	48	<95	<76	<58	<10	10
JPN-B-3	8.0	20	<55	51	180	<76	590	<10	14
MMR-B-1	5.0	28	<55	39	130	<76	380	<10	22
SGP-B-1	6.0	<3.6	<55	54	280	<76	560	<10	71
THA-B-1	23	<3.6	<55	52	1500	<76	1300	48	65
THA-B-2	5.0	<3.6	<55	<2.1	<95	<76	1200	28	6.0
VNM-B-1	22	36	<55	66	230	<76	540	<10	190
**Europe (*n* =11)**									
BEL-B-1	12	<3.6	<55	140	230	<76	490	<10	63
BEL-B-2	9.0	21	<55	130	240	<76	660	9600	220
DEU-B-1	10	33	<55	290	240	<76	780	30	220
ESP-B-1	8.0	<3.6	<55	22	<95	<76	<58	<10	55
ESP-B-2	9.0	20	<55	<2.1	140	<76	<58	<10	410
NOR-B-1	4.0	<3.6	<55	<2.1	<95	<76	420	<10	18
RUS-B-1	12	<3.6	<55	280	320	<76	570	<10	280
SWE-B-1	6.0	<3.6	<55	37	110	<76	630	<10	220
SWE-B-2	8.0	<3.6	<55	13	<95	<76	450	<10	150
UK-B-1-	10	<3.6	<55	16	120	<76	510	<10	110
UK-B-2-	<2.9	<3.6	120	<2.1	<95	<76	1000	<10	73
**North America (*n* =4)**									
MEX-B-1	9.0	<3.6	<55	62	<95	<76	1100	300	120
USA-B-1	8.0	<3.6	<55	19	120	<76	860	<10	380
USA-B-2	5.0	<3.6	<55	50	190	<76	2300	28	520
USA-B-3	5.0	22	<55	<2.1	<95	<76	2000	<10	170
**Lid**									
**Asia (*n* =12)**									
CHN-B-1	<2.9	<3.6	<55	<2.1	110	<76	390	<10	11
CHN-B-2	4.0	<3.6	<55	7.0	<95	<76	1700	<10	6.0
CHN-B-3	9.0	14	<55	<2.1	<95	<76	430	<10	8.0
IDN-B-1	4.0	<3.6	<55	<2.1	<95	<76	710	<10	5.0
JPN-B-1	6.0	12	<55	<2.1	<95	<76	160	<10	7.0
JPN-B-2	7.0	<3.6	<55	<2.1	<95	<76	210	<10	11
JPN-B-3	13	<3.6	<55	14	160	<76	690	<10	7.0
MMR-B-1	9.0	20	<55	12	96	<76	210	<10	14
SGP-B-1	6.0	<3.6	<55	17	<95	<76	400	<10	19
THA-B-1	11	<3.6	<55	15	<95	<76	260	<10	7.0
THA-B-2	4.0	<3.6	<55	<2.1	<95	<76	<58	12	<4.5
VNM-B-1	7.0	18	<55	<2.1	<95	<76	210	<10	12
**Europe (*n* =11)**									
BEL-B-1	5.0	<3.6	<55	<2.1	<95	<76	<58	<10	<4.5
BEL-B-2	10	<3.6	<55	<2.1	<95	110	230	<10	9.0
DEU-B-1	10	23	<55	19	<95	<76	<58	<10	48
ESP-B-1	4.0	<3.6	<55	<2.1	<95	<76	<58	<10	8.0
ESP-B-2	7.0	<3.6	<55	<2.1	<95	<76	320	<10	11
NOR-B-1	5.0	<3.6	<55	8	<95	<76	5300	<10	10
RUS-B-1	3.0	<3.6	<55	<2.1	<95	<76	270	<10	6.0
SWE-B-1	5.0	15	<55	20	<95	<76	640	<10	110
SWE-B-2	8.0	14	<55	16	<95	<76	330	<10	12
UK-B-1	10	<3.6	<55	<2.1	99	<76	200	<10	31
UK-B-2	<2.9	<3.6	<55	<2.1	<95	<76	570	<10	57
**North America (*n* =4)**									
MEX-B-1	7.0	<3.6	<55	<2.1	<95	<76	750	<10	15
USA-B-1	10	<3.6	<55	<2.1	<95	<76	430	<10	19
USA-B-2	6.0	<3.6	<55	<2.1	<95	<76	1400	<10	110
USA-B-3	8.0	24	<55	<2.1	<95	<76	360	<10	220

DMP: Dimethyl Phthalate; DEP: Diethyl phthalate; DAP: Diallyl phthalate; DiBP: Diisobutyl phthalate; DBP: Dibutyl phthalate; BBP: Benzyl butyl phthalate; DEHP: Bis(2-ethylhexyl) phthalate; DOA: Dioctyl adipate; BHT: Butylated hydroxytoluene.

Table S6. Eigenvalues of the PCA for additives correlations

Eigenvector(s)	PC 1	PC 2
Dimethyl phthalate (DMP)	1.48	-1.72
Diethyl phthalate (DEP)	-5.28	-2.67
Diisobutyl phthalate (DiBP)	-1.49	1.48
Dibutyl phthalate (DBP)	-0.16	1.61
Bis(2-ethylhexyl) phthalate (DEHP)	3.30	-0.47
Dioctyl adipate (DOA)	-0.52	3.85
Butylated hydroxytoluene (BHT)	2.67	-2.08
